# Progression rate in Bosniak category IIF complex renal cysts

**DOI:** 10.1590/0100-3984.2018.0038

**Published:** 2019

**Authors:** Amanda de Vasconcelos Chambi Tames, Eduardo Kaiser Ururahy Nunes Fonseca, Fernando Ide Yamauchi, Gabriela Maia Soares Messaggi Arrais, Thais Caldara Mussi de Andrade, Ronaldo Hueb Baroni

**Affiliations:** 1 Hospital Israelita Albert Einstein, Department of Radiology and Diagnostic Imaging, São Paulo, SP, Brazil.

**Keywords:** Tomography, X-ray computed, Magnetic resonance imaging, Cysts/classification, Carcinoma, renal cell, Kidney/diagnostic imaging, Tomografia computadorizada, Ressonância magnética, Cistos/classificação, Carcinoma de células renais, Rim/diagnóstico por imagem

## Abstract

**Objective:**

To evaluate progression rate of Bosniak category IIF complex renal cysts and the malignancy rate among surgically resected cysts.

**Materials and Methods:**

We performed a database search for complex renal cysts classified as Bosniak category IIF on computed tomography or magnetic resonance imaging between January 2008 and April 2016. Follow-up examinations (computed tomography or magnetic resonance imaging) were used in order to evaluate progression (Bosniak category reclassification) and stability, the latter being defined as remaining stable for a minimum of six months. Pathology reports were used as the reference to assess the malignancy rate of surgically resected cysts.

**Results:**

A total of 152 cysts in 143 patients were included in the final analysis. Seven cysts (4.6%) were reclassified on follow-up studies, and mean time to progression was 20 months (range, 1 month to 4 years). Three cysts were surgically resected. All three were diagnosed as low-grade malignant renal cell carcinomas (RCCs): one clear cell RCC and two papillary RCCs. The remaining 145 cysts remained unchanged after a mean follow-up period of 28 months (range, 6 to 118 months).

**Conclusion:**

The progression rate in Bosniak category IIF cysts was low. Even lesions that were upgraded on follow-up remained stable, indicating an indolent behavior. Our data support the idea of conservative management of Bosniak IIF renal cyst.

## Abstract (pt)

**Objetivo:**

Avaliar a taxa de progressão das lesões císticas renais complexas Bosniak II-F e a taxa de neoplasia maligna nas lesões ressecadas cirurgicamente.

**Materiais e Métodos:**

Realizamos uma pesquisa no banco de dados da nossa instituição reunindo lesões císticas renais complexas classificadas como Bosniak II-F em exames de tomografia computadorizada ou ressonância magnética, de janeiro de 2008 a abril de 2016. Foram utilizados exames de acompanhamento (tomografia computadorizada ou ressonância magnética) para avaliar a progressão na categoria Bosniak, com um mínimo de seis meses de estabilidade. Os relatórios de patologia foram utilizados como referência para avaliar a taxa de neoplasia maligna de lesões ressecadas cirurgicamente.

**Resultados:**

Um total de 152 lesões em 143 pacientes foi incluído na análise final. Sete lesões foram reclassificadas em estudos de acompanhamento (4,6%) e o tempo médio de progressão foi de 20 meses (1 mês a 4 anos). Três pacientes tiveram cistos ressecados cirurgicamente, todos diagnosticados como carcinoma de células renais: um do subtipo células claras e dois papilíferos, todos de baixo grau. As 145 lesões restantes permaneceram inalteradas, com seguimento médio de 28 meses (6 a 118 meses).

**Conclusão:**

A taxa de progressão nos cistos categoria Bosniak II-F foi baixa. Mesmo as lesões que tiveram *upgrade* da categoria de Bosniak no seguimento permaneceram estáveis, indicando um comportamento indolente. Nossos dados apoiam a ideia de tratamento conservador do cisto renal Bosniak II-F.

## INTRODUCTION

Renal cysts are the most common incidental finding in the daily practice of radiology, with an estimated prevalence of 50% in patients over 50 years of age^(^^1^^-^^4^^)^. Most renal cysts are acquired lesions, are asymptomatic, and are incidentally diagnosed on routine tests such as ultrasound, computed tomography (CT), and magnetic resonance imaging (MRI).

The majority of renal cysts are benign. Simple cysts are easily characterized by routine imaging methods and do not require histopathological analysis. However, certain features, such as septa, wall thickening, areas of enhancement, and mural nodules, increase the complexity of a cyst and consequently the risk of malignancy. In patients with complex renal cysts, CT and MRI play an important role, not only in the characterization of the cysts but also in guiding their appropriate management.

The Bosniak classification system, created in 1986, established morphological CT criteria to differentiate between cysts that are probably benign and those that are likely to be malignant^(^^1^^-^^8^^)^. To standardize communication between radiologists and urologists, Bosniak initially described four categories. Cysts classified as category I or II were considered presumably benign, therefore not requiring follow-up or surgical management; those classified as category III or IV were considered to have an increased risk of malignancy, therefore requiring surgical management^(^^1^^-^^8^^)^. In 1993, Bosniak introduced a new category (IIF), in an attempt to stratify category III cysts, category IIF being applied to those that were likely benign but more complex than category II cysts^(^^2^^)^. Minimally complicated cysts, such as those containing numerous thin septa or thick calcifications, and hyperattenuating cysts that are completely parenchymal and larger than 3.0 cm were included in the IIF category, therefore requiring follow-up to confirm their stability^(^^9^^-^^11^^)^.

The Bosniak classification system has been widely used by radiologists and urologists, having recently been found to be also suitable for use in the evaluation of renal cysts on MRI scans^(^^7^^,^^11^^-^^14^^)^. Although there have been many studies evaluating the malignancy rates of category III and IV cysts^(^^2^^,^^4^^,^^6^^,^^7^^)^, few studies have evaluated the malignancy rates and progression of category IIF cysts. Previous studies of category IIF cysts have had small sample sizes^(^^3^^,^^5^^)^, have involved short follow-up periods^(^^5^^)^, or have produced discrepant results^(^^1^^-^^5^^)^. Therefore, the objective of the present study was to evaluate Bosniak category IIF cysts, in terms of their progression and the malignancy rate after surgical resection, in a large cohort of patients.

## MATERIALS AND METHODS

This was a single-center retrospective study. We searched the radiology database of our institution for CT or MRI reports that contained the term "Bosniak IIF" and were issued between January 2008 and April 2016. The study was approved by the institutional review board. Because of the retrospective nature of the study, the requirement for written informed consent was waived.

Examinations were prospectively read in consensus by two board-certified abdominal radiologists with 3 to 20 years of experience, and all cases included in this study were reviewed by one of the authors. Discrepant cases were reviewed in consensus with a senior author with 15 years of experience in abdominal radiology. For image interpretation, cases were read on an integrated Picture Archiving and Communication System and radiology information system that included multiplanar reconstruction tools (Carestream Health; Rochester, NY, USA).

### Imaging technique

CT examinations were performed in several different 16- to 32-slice multidetector CT scanners, with a collimation of ≤ 2 mm, including protocols for routine abdominal imaging, abdominal pain, renal masses, and urography, all of which involved, at a minimum, an unenhanced phase and a contrast-enhanced venous phase (i.e., a 70-s delay) or nephrographic phase (i.e., a 90-s delay). Iodinated contrast medium (1-2 mL/kg of body weight) was delivered intravenously via a power injector at a rate of 2.0-3.0 mL/s.

MRI examinations were performed in several different 1.5 or 3.0 T scanners. At a minimum, axial and coronal fast spin-echo T2-weighted images, chemical shift-based images, diffusion-weighted images, and unenhanced and contrast-enhanced T1-weighted sequences were obtained, in all cases (including corticomedullary, nephrographic, and excretory phases), of the area extending from the diaphragm to below the inferior renal pole. Gadolinium-based contrast medium (0.2 mL/kg of body weight) was injected at a rate of 2 mL/s.

### Inclusion and exclusion criteria

The inclusion criterion was having renal cysts that were classified as Bosniak IIF on CT or MRI and were reclassified on a subsequent examination or demonstrated stability for at least 6 months in follow-up studies. The exclusion criteria were severe artifacts degradation on imaging examinations, cysts smaller than 1.0 cm, and follow-up studies of stable cysts performed after less than 6 months.

Our initial search generated 428 reports of Bosniak category IIF cysts. Of those 428 reports, 276 were excluded, for the following reasons: no follow-up examinations (n = 233); cyst size < 1.0 cm (n = 19); and a follow-up period shorter than 6 months (n = 24).

### Cyst progression and follow-up

Progression was defined as the appearance of new worrisome characteristics in a cyst, leading to its reclassification as Bosniak category III or IV^(^^1^^-^^8^^)^. The first follow-up examination demonstrating such reclassification was considered a reference point for determining the time to progression. If conservative management was chosen, the latest available follow-up study was considered in order to evaluate stability. If surgery was performed, pathological reports were evaluated. None of our category IIF cysts without progression were submitted to biopsy or surgery. During the follow-up period, none of those cysts were reclassified as being in a lower category.

The initial imaging modality employed was MRI in 88 patients (65 follow-up examinations with MRI and 23 follow-up examinations with CT) and CT in 64 patients (21 follow-up examinations with MRI and 43 follow-up examinations with CT).

## RESULTS

**Figure 1:**
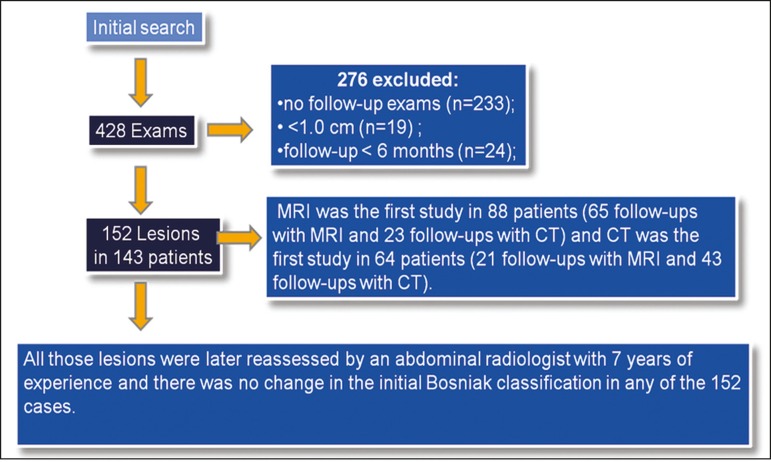
Flow chart of the patient selection process, with exclusion criteria.

The final sample included 152 Bosniak category IIF cysts in 143 patients (7 patients had two cysts, and 1 patient had three cysts), as depicted in Figure 1. The mean cyst size in the first examination was 3.0 cm (range, 1 to 16 cm), and the mean follow-up period was 28 months (range, 6 to 118 months), as shown in Table 1.

**Table 1:** Characteristics of the cysts/patients included in the study.

Characteristic	Values
Number of cysts	152
Number of patients	143
Patient age (years), range (mean)	31-94 (63)
Patient gender, n (%)	
Male	107 (74.8)
Female	36 (25.2)
Cyst laterality	
Right	78 (51.3)
Left	74 (48.7)
Cyst size (cm), range (mean)	1.0-16.0 (3.0)
Follow-up (months), range (mean)	6-118 (28)
Progression, number of cysts	7

During the follow-up period, seven (4.6%) of the Bosniak category IIF cysts were reclassified: two as category III and five as category IV. The mean size of the reclassified cysts was 1.9 cm (range, 1.4 to 2.7 cm), and the mean time to progression was 20 months (range, 1 month to 4 years): two cysts showed progression before 6 months of follow-up (by 1 and 3 months, respectively); four cysts showed progression between months 11 and 36; and one cyst showed progression after 48 months of follow-up. The mean growth rate of the reclassified cysts was 0.04 cm (range, −0.7 to 1.1 cm): the size of one cyst increased by 1.1 cm; and two cysts showed a reduction in size but developed new worrisome features (one developed a hypervascular mural nodule-therefore being reclassified as category IV-and the other showed multiple thick septa-therefore being reclassified as category III). Table 2 summarizes the characteristics of the reclassified cysts.

**Table 2:** Detailed characteristics of the seven Bosniak category IIF cysts that showed progression and were reclassified.

Patient	Time to progression (months)	Initial size (cm)	Final size (cm)	Size variation (cm)	Initial imaging modality	Follow-up imaging modality	New features	Final Bosniak category	Outcome	Age at the progression examination (years)
1	46	1.3	2.4	1.1	CT	MRI	Solid component	IV	Surgery (papillary RCC)	50
2	11	1.4	1.5	0.1	MRI	MRI	Solid component	IV	Surgery (papillary RCC)	33
3	3	1.7	1.7	0	MRI	MRI	Thick septa with post-contrast enhancement	III	Follow-up*	54
4	26	2.4	1.8	-0.6	CT	MRI	Solid component	IV	Follow-up†	86
5	1	2.7	2.7	0	CT	MRI	Solid component	IV	Lost to follow-up	32
6	48	2.8	2.1	-0.7	CT	MRI	Thick walls and septa	III	Lost to follow-up	74
7	15	1	1.4	0.4	MRI	CT	Solid component	IV	Surgery (clear cell RCC)	47

* Patient 3 had comorbidities (cirrhosis with hepatocellular carcinoma) and therefore did not undergo surgery.

† Patient 4 decided not to undergo surgery because of his advanced age and comorbidities.

Of the seven reclassified cysts, three were surgically resected at our facility. Of those three cysts, all of which had been reclassified as Bosniak category IV, one was found to be a clear cell renal cell carcinoma (RCC) and two were found to be papillary RCCs. The postoperative follow-up period ranged from 16 months to 30 months (mean, 24 months), and there was no evidence of recurrence. Two of those seven patients were followed clinically: one was 86 years old and had a Bosniak category IV cyst that remained stable after one year of follow-up; and the other had cirrhosis and hepatocellular carcinoma and a Bosniak category III cyst that also remained stable after one year of follow-up. The last two patients with category IIF cysts that were reclassified (to category IV and category III, respectively) were lost to follow-up.

The remaining 145 Bosniak category IIF cysts remained unchanged on follow-up studies and were not reclassified. The growth rate of those cysts ranged from 0.1 cm to 4.1 cm (mean, 0.3 cm), one having grown more than 4 cm over 6 years, without significant changes in morphology. The mean follow-up was 28 months (range, 6 to 118 months).

**Figure 2:**
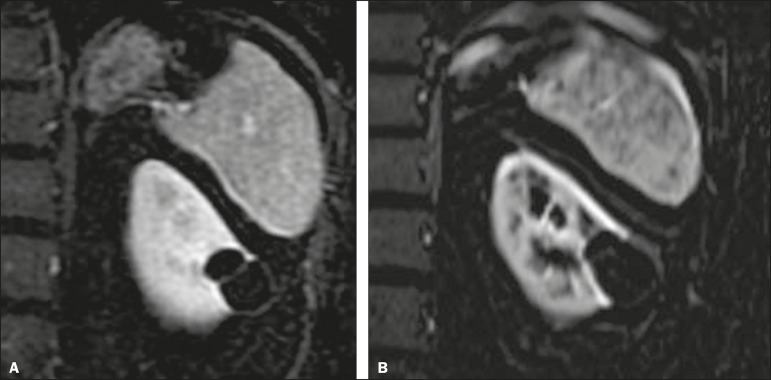
Upper abdominal MRI, in a coronal view after digital subtraction, showing a cyst in the middle of the left kidney, containing some septa and presenting mild enhancement after gadolinium injection (Bosniak category IIF). Although the cyst presented minimal growth between 2007 (**A**) and 2012 (**B**), its characteristics remained stable and it remained a Bosniak IIF lesion.

**Figure 3:**
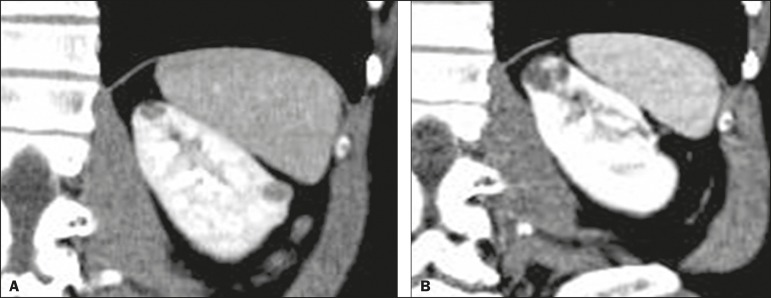
Abdominal CT scan, in a coronal view after intravenous contrast injection, showing a cyst in the upper pole of the left kidney, with some septa and minimal internal enhancement (Bosniak category IIF) in 2011 (**A**). A follow-up CT scan of the same patient in 2015 (**B**) demonstrated not only a significant increase in the size of the cyst, but also a mural nodule and an increase in the thickness of its septa (the cyst being reclassified as category IV). The cyst was surgically excised, and the final diagnosis was papillary carcinoma.

Comparisons between the stable and reclassified cyst groups are summarized in Table 3. Figures 2 and 3, respectively, illustrate a cyst that remained stable and a cyst that was reclassified.

**Table 3:** Comparison between the Bosniak category IIF cysts that progressed (i.e., were reclassified) and those that remained stable.

	Progression	
Characteristics	No (n = 145)	Yes (n = 7)
Age (years), range (mean)	31-94 (63)	36-86 (57)
Male/female, n/n	100/36	7/0
Size (cm), range (mean)	1.0-16 (3.1)	1.0-2.8 (1.9)
Time to progression (months), range (mean)	6-118 (28)	1-48 (20)

## DISCUSSION

The Bosniak system for classifying renal cysts into five categories is well established, and the specific management strategies recommended for each category have been widely adopted^(^^1^^-^^8^^)^. For category I and II cysts, no specific recommendation is made, because such cysts are presumably benign. For category III and IV cysts, surgery is often considered, given the high risk of malignancy, although some authors believe they are less aggressive than are non-cystic renal neoplasms^(^^2^^,^^4^^,^^5^^,^^15^^)^. For category IIF cysts, the length of follow-up required is still under debate. The American College of Radiology suggests CT or MRI follow-up examination every 6-12 months for up to 5 years after the initial diagnosis^(^^2^^)^. The revised Bosniak system proposed that IIF cysts with fewer complex features (i.e., more similar to category II cysts) could be followed for two years after initial diagnosis, whereas those that were more complex (i.e., more similar to category III cysts) should be followed for up to four years^(^^7^^)^. The authors of another study suggested that, in addition to morphological features, patient age should be taken into consideration when deciding how long the follow-up period should be^(^^16^^)^: five years for patients 50-60 years of age and longer for younger patients.

A recent systematic review and meta-analysis^(^^17^^)^ addressed the Bosniak classification in CT in terms of its diagnostic performance and the malignancy rates for the various categories. The review showed that malignancy rates were higher in studies that used histopathology alone as the reference standard than in those that used a combination of histopathology and clinical follow-up. In the present study, the rate of progression for Bosniak category IIF cysts was 4.6%, which is slightly lower than that reported in previous studies^(^^1^^-^^5^^)^, including a recent study that reported a rate of 10.9%^(^^2^^)^. However, in the present study, the histopathology showed that all of the resected cysts were malignant RCCs.

Of the seven cysts that were reclassified in our study, two were reclassified within the first six months of follow-up, and the five remaining cysts were reclassified between month 11 to month 48. One of the two rapidly progressing cysts had shown thin septa on the initial MRI and measurable septal enhancement on a subsequent MRI, therefore being reclassified as Bosniak category III. The other fast progressing cyst was likely related to higher contrast resolution of MRI when compared to CT. In the initial examination (by CT), it appeared as a 2.7 cm hyperattenuating cyst with questionably thickened walls classified as category IIF. An MRI scan one month later showed a small mural nodule and the cyst was therefore reclassified as category IV. As previously reported, multiple septa, septal thickening, and small solid components are more evident on MRI scans than on CT scans^(^^7^^,^^12^^-^^14^^,^^17^^)^. Particularly hyperattenuating or heavily calcified on CT may benefit from MRI, given the higher contrast resolution for tissue characterization on T2-weighted images and post-contrast sequences with digital subtraction^(^^7^^,^^14^^,^^15^^,^^18^^)^. For both of the cysts mentioned above, what appeared to be rapid progression was more likely related to their reclassification on MRI rather than to true progression. We believe that those lesions were already category III/IV cysts, and their reclassification being secondary to an initial underestimation of the septal enhancement in one and to better characterization of the small nodule in the subsequent MRI of the other. Despite the short time to progression in both of those cysts, no metastases were seen in the follow-up studies. In fact, cystic neoplasms presenting as category IIF are more likely to be less aggressive neoplasms than to be conventional RCCs^(^^19^^-^^23^^)^. Two of the cysts that were reclassified in our study (one as category III and one as category IV) were managed through conservative follow-up, because both patients had comorbidities and one was of advanced age, both cysts remaining stable at 12 months. Three of the seven reclassified cysts were treated surgically; all three were subsequently identified as malignant RCCs, although they were early stage, low-grade RCCs.

Our study has some limitations. First, the majority of the Bosniak category IIF cysts identified were excluded due to lack of follow-up, which could have introduced a selection bias. Second, stability during follow-up is not necessarily indicative of benignity. Third, there were very few cases in which there was histological confirmation of malignancy, which precluded a more detailed analysis of progression rates.

## CONCLUSION

The progression rate in Bosniak category IIF cysts was low. Even lesions that were upgraded on follow-up remained stable, indicating an indolent behavior. Our data support the idea of conservative management of Bosniak IIF renal cyst.
